# Copper sulfate induces clinico-hematological, oxidative stress, serum biochemical and histopathological changes in freshwater fish rohu (*Labeo rohita*)

**DOI:** 10.3389/fvets.2023.1142042

**Published:** 2023-03-09

**Authors:** Saima Naz, Riaz Hussain, Zhang Guangbin, Ahmad Manan Mustafa Chatha, Zia Ur Rehman, Shfaq Jahan, Momil Liaquat, Ahrar Khan

**Affiliations:** ^1^Department of Zoology, Government Sadiq College Women University, Bahawalpur, Punjab, Pakistan; ^2^Department of Pathology, Faculty of Veterinary and Animal Sciences, The Islamia University of Bahawalpur, Bahawalpur, Pakistan; ^3^Shandong Vocational Animal Science and Veterinary College, Weifang, China; ^4^Department of Entomology, Faculty of Agriculture and Environment, The Islamia University of Bahawalpur, Bahawalpur, Pakistan; ^5^Department of Physiology, Faculty of Veterinary and Animal Sciences, The Islamia University of Bahawalpur, Bahawalpur, Pakistan; ^6^Faculty of Veterinary Science, University of Agriculture, Faisalabad, Pakistan

**Keywords:** copper sulfate, *Labeo rohita*, blood-biochemistry, oxidative stress, histopathology

## Abstract

Despite being an essential trace element for numerous metabolic processes and micronutrients, copper (Cu) has induced adverse effects on the environment and public health due to its continuous and widespread use for the last several decades. The current study assessed the hematological and histopathological alterations in the freshwater fish (*Labeo rohita*) exposed to graded concentrations of copper sulfate. For this purpose, *L. rohita* fish (*n* = 72), weighing ~200–215 g, were randomly divided into four experimental groups and then exposed to acute doses of CuSO_4_, i.e., control, 0.28, 0.42, and 0.56 μgL^−1^. For comparative analysis of hematological and biochemical changes, blood/serum samples were obtained on 12, 24, and 36 days. Overall, the body weight of fish decreased with the time and dose of CuSO_4_; as the dose increases, body weight decreases. Dose and time-dependent results were observed in other parameters also. Results showed a significant increase in leukocytes, whereas red blood cells count, Hb, and Hct were significantly reduced in treated groups compared to the control. The mean corpuscular hemoglobin (MHC) and mean corpuscular hemoglobin concentration (MCHC) showed a non-significant decrease in treated groups compared to the control group. Serum biochemical parameters, including total proteins, albumin, and globulin, decreased significantly (*p* < 0.05). At the same time, alanine aminotransferase (ALT), aspartate aminotransferase (AST), alkaline phosphatase (ALP), lactate dehydrogenase (LDH), glucose, and cholesterol were significantly (*p* < 0.05) increased in the treated groups compared to the control group. Significantly (*p* < 0.05) increased levels of lipid peroxidation while decreased values of antioxidant enzymes, including superoxide dismutase (SOD), catalase (CAT), and reduced glutathione (RGSH) in the blood of fish were recorded. Histopathological examination of fish gills, liver, and kidneys showed inflammation and degenerative changes due to CuSO_4_ exposure. In the brain tissue, degenerative changes like neuron necrosis, intracellular edema, cytoplasmic vacuolization, and congestion were observed. In conclusion, the study indicates that exposure to copper sulfate, even in smaller concentrations, can cause adverse hematological and histopathological changes in *L. rohita* fish.

## Introduction

Increased agricultural activities and extensive industrialization ultimately enhance the levels of various metals in the aquatic environment. Copper (Cu), an essential micronutrient for humans and animals, is an important and vital trace element needed for the multiple normal metabolic functions in various living organisms (1, 2). Copper also has an essential role in the regular activity of connective tissue, iron metabolism, and several functions in the central nervous system (3, 4). Plants and animals need copper to perform everyday tasks in different organs, e.g., hemoglobin synthesis. It is also a cofactor for various enzymes (5–7).

Widespread application of copper sulfate (CuSO_4_) in various sectors like building materials, agricultural sector, electronic industry, water pipes, transportation sector, intra-uterine contraceptive devices, and as an antifungal agent and growth promoter in poultry has also induced toxicological impacts in the exposed species (8). Copper sulfate is used worldwide as a fungicide in agriculture and as an algaecide in aquaculture (9). Unlike other synthetic and organic pesticides, Cu cannot be degraded and can easily be deposited in various tissues of the treated animals (8, 10, 11). Due to frequent application, Cu can damage the cells due to its potential to catalyze the generation of reactive oxygen species (12). Copper sulfate (CuSO_4_) negatively affected male Nile tilapia's reproductive function (13). Studies have reported that persistent dietary and environmental Cu exposure may cause different neurological and reproductive disorders and hepatic diseases in various animals (8, 14, 15). Cu also causes toxic impacts in terms of poor feed intake, increased oxidative stress, reduced body mass, and biochemical and hematological changes (5, 8, 16).

Over the last years, aquatic contamination has become a great concern for marine life and public health (17–19). Anthropogenic activities and accidental industrial waste discharge have become major causes of aquatic pollution in freshwater (20). Pollution due to heavy metals occur in the aquatic ecosystem due to agricultural discharge, geological weathering, municipal, direct air deposition, and other industrial waste products (21–24). Due to anthropogenic activities, several toxicants, including CuSO_4_, enter the aquatic ecosystem and cause detrimental effects on the exposed aquatic organisms (13). Fishes are considered an important component of the aquatic food chain among different aquatic organisms and reflect almost all the adverse effects upon exposure to toxicants (25–28). High pollution due to various heavy metals in water has become a significant problem because of the threat to aquatic life and public health concerns related to fish consumption (23, 29, 30). In the natural environment and laboratory specimens, various metals have been linked with different fish abnormalities (31). Constant subjection to several metals, like Zn, Cu, Pb, Cd, Ni, and As, causes harmful effects in organisms (32).

Biochemical parameters are often considered indicators when a clinical diagnosis of aquatic organisms' physiology is applied to determine the effects of external stressors and toxic substances (33). For the contaminants, cellular enzymes (33) and biochemical and oxidative stress parameters (34–40) serve as a biomarker for assessing the risk of environmental hazards (41). The microscopic evaluation of target tissues using histopathological examination is the endpoint in determining the risks of contaminants in the environment (42). Histopathological alterations can be employed to analyze the effects of numerous environmental contaminants on organisms as well as the general health of the aquatic population (43, 44). Investigation of biochemical parameters is recommended to give early indicators regarding modifications in the organisms exposed to toxicants and is especially effective in identifying target organs with toxicity and animal health (26, 45). Thus, the objectives of this study were to ascertain the toxicological impact of CuSO_4_ in *Labeo rohita* along with dose and time-dependent manner so that a suitable safe, and eco-friendly dose of CuSO_4_ could be determined for the removal of various contaminants from fish pounds.

## Materials and methods

### Experimental species, management, and treatments

A total of 72 freshwater fish *L. rohita*, F. Hamilton, 1822, healthy and free from any clinical ailments, with approximately the same age and weight (200–215 g), were collected from a local fish hatchery and breeding center. All the fish were immediately transported to the laboratory and reared in glass aquaria having 100 L of water at 26 ± 1.0 °C and photo-regime (12/12 h) light/dark for 2 weeks under laboratory conditions. Before the start and during the trial, all the fish were offered Optimum Aquarium Fish Food having 28% crude proteins (Perfect Companion, Thailand). The feed was provided to all the fish at 2–3% of body mass twice daily. After 2 weeks, the fish were divided randomly into equal four groups (A-D) having 18 fish in each group. Copper sulfate (CuSO_4_) of analytical grade was procured from the local market. The concentrations of CuSO_4_ were selected following the previous studies (46, 47). Briefly, CuSO_4_ was dissolved in water @ 0.28, 0.42, and 0.56 μg.L^−1^, respectively and fish in groups B-D were treated with the prepared solution for 36 days.

### Physical parameters

Clinical and behavioral signs include loss of equilibrium, mucus secretion from mouth and gills, air gulping, increased swimming, rapid operculum movement, bulging eyes, loss of coordination, erratic swimming, and swimming in isolation in fish treated with various concentrations of copper sulfate were estimated numerically roughly. Each sign was given score – to +++ (– means no sign while + to +++ denoted mild, moderate, and severe, respectively (48). Data thus collected were averaged and presented.

### Blood sampling and estimation of hemato-biochemical parameters

On days 12th, 24th, and 36th post-treatment, ~2.0 mL of blood from the caudal vein of six randomly selected fish was collected using a 26-gauge hypodermic sterile needle after the fish was anesthetized with clove oil (4 mg.L^−1^) (27). A blood sample was placed in anticoagulant-coated glass test tubes on each collection day. Various hematological parameters, including hemoglobin (Hb) quantity, hematocrit (Hct), red blood cells (RBCs) counts, total and differential white blood cells (WBCs) counts, lymphocytes, and neutrophils were determined as described previously (28). Total proteins (Cat # 997180) were assessed according to the earlier method (27). After centrifugation, serum was separated and stored at −20°C. Serum biochemical parameters like aspartate aminotransferase (AST; Cat # 30243), alanine aminotransferase (ALT; Cat # 30254), creatinine (Cat # 99108), cholesterol (Cat # 30183), alkaline phosphatase (ALP; Cat # 30134) and triglycerides (Cat # 30364) were measured using commercial kits (Canovelles; Barcelona, Spain). The quantity of lactate dehydrogenase (LDH) was measured by using a commercial kit (Cat # 21213; Analytical, Germany), while the quantity of urea (Cat # 996060) and albumin (Cat # 997258) was measured with the help of commercially available kits (Quimica Clinica Aplicada, S.A. Spain) using chemistry analyzer (Model: Rx Monza # 328-15-1001; UK) at days 12th, 24th and 36th of the trial (27).

### Body mass, absolute and relative organ weight, and histopathology

Randomly selected, six fish were weighed on the 12th, 24th, and 36th day of the trial, then an autopsy was conducted. Visceral organs like the kidneys, brain, gills, and liver were dissected and weighed immediately. The relative weight was measured as a percent of the body weight. Similarly, the liver, kidneys, brain, and gills were preserved in 10% paraformaldehyde for microscopic studies. After fixation, all the visceral tissues were processed for histopathological changes using eosin and hematoxylin staining procedures (27). Tissue slides were examined for the presence of lesions by two pathologists, and if discrepancy come across, then a third pathologist was asked for judgment. Thus, collected histopathological lesions were graded by earmarking numerics. All the slides in each group were studied; median of the numerics was calculated and an cumulative picture of each lesion in each group was drawn. The severity/degree of different histopathological lesions were indicated on the basis of scoring of lesions as mild (25%), moderate (50%), severe (75%) and very severe (100%).

### Status of oxidative stress parameters and antioxidant enzymes in erythrocytes

TBARS values were determined according to the procedure already described (49), while reduced glutathione (RGSH), superoxide dismutase (SOD), and catalase (CAT) were determined according to modified methods of Beutler et al. (50), Marklund and Marklund (51) and Chance and Maehly (52), respectively.

### Data analysis

The data thus collected from hemato-biochemical, oxidative stress, and antioxidant enzymic parameters were presented as means ± standard error (SE). The data were subjected to ANOVA (one-way analysis of variance) using statistical software (IBM SPSS Statistics, v.22.00) followed by *post hoc* Tukey's test to determine the significant variation at *p* < 0.05.

## Results

### Physical observations

The present investigation observed no mortality in the treatment groups (B, C, D) during the trial. Clinical and behavioral signs include loss of equilibrium, mucus secretion from mouth and gills, air gulping, increased swimming, rapid operculum movement, bulging eyes, loss of coordination, erratic swimming, and swimming in isolation detected in the treated fish. The severity of various clinical and behavioral signs increases parallelly with increased concentration and exposure time to CuSO_4_ (Table 1).

**Table 1:** Intensity of various clinical signs exhibited by the *Labeo rohita* treated with various concentrations of copper sulfate.

**Clinical ailments**	**Groups/treatments**		
	**B (0.28** μ**g.L**^−1^**)**	**C (0.42** μ**g.L**^−1^**)**	**D (0.56** μ**g.L**^−1^**)**
Loss of equilibrium	– – +	– + +	+ + +
Mucus secretion from mouth and gills	– – +	– + +	+ + +
Air gulping	– – +	– + +	+ + + +
Increased swimming	– +	– + +	+ + +
Rapid operculum movement	– +	– + + +	+ + + +
Bulging eyes	– +	+ + +	+ + +
Coordination loss	– – +	– + +	+ + +
Erratic swimming	– +	+ + +	+ + + +
Swimming in isolation	– +	+ + +	+ + +
Average	Mild	Moderate	Severe

Fish kept in group A were kept as control and showed no clinical ailments. Copper sulfate was dissolved in sterile water and the fish in groups B to D were treated to 0.28, 0.42, and 0.56 μg.L^−1^, respectively for 36 days.

### The body weight and absolute weight of various visceral tissues of *Labeo rohita*

Results showed that the body weight of fish decreased significantly (*p* < 0.05) in group D (CuSO_4_: 0.56 μgL^−1^) compared to the control group on the 36th experimental day (Table 2). The absolute weight of the brain, kidneys, and liver increased gradually at CuSO_4_ doses of 0.28 μgL^−1^ and 0.48 μgL^−1^ on experimental days 12 and 24. In contrast, at the CuSO_4_ dose of 0.56 μgL^−1^, the absolute weight of these organs increased significantly (*p* < 0.05) than the control group on experimental days 12, 24, and 36 (Table 2). Moreover, the absolute weight of the kidneys and liver also increased significantly (*p* < 0.05) in the control group at CuSO_4_ dose 0.48 μgL^−1^ on experimental day 36. The absolute weight of gills increased significantly (*p* < 0.05) at CuSO_4_ dose 0.56 μgL^−1^ than the control group on the 36th experimental day (Table 2).

**Table 2:** Body weight and absolute weight of various visceral organs of *Labeo rohita* treated to copper sulfate with different doses.

**Parameters/day**	**Groups/treatments**			
	**A (0.0)**	**B (0.28** μ**g.L**^−1^**)**	**C (0.42** μ**g.L**^−1^**)**	**D (0.56** μ**g.L**^−1^**)**
**Body weight (g)**				
12	166.0 ± 2.04	164.25 ± 3.81	160.75 ± 3.42	156.5 ± 1.84
24	168.5 ± 4.36	162.75 ± 3.03	162.5 ± 3.27	158.75 ± 2.28
36	169.25 ± 4.21	162.25 ± 1.95	158.0 ± 1.65	155.75 ± 4.21^*^
**Absolute weight of brain (g)**				
12	0.71 ± 0.27	0.72 ± 0.26	0.75 ± 0.21	0.98 ± 0.20^*^
24	0.74 ± 0.26	0.73 ± 0.01	0.76 ± 0.24	0.99 ± 0.29^*^
36	0.71 ± 0.25	0.74 ± 0.23	0.77 ± 0.25	0.99 ± 0.30^*^
**Absolute weight of gills (g)**				
12	4.65 ± 0.46	4.82 ± 0.51	5.05 ± 0.45	5.15 ± 0.39
24	5.05 ± 0.42	5.01 ± 0.49	5.06 ± 0.90	5.59 ± 0.87
36	5.06 ± 0.42	5.29 ± 0.38	5.57 ± 0.48	7.82 ± 0.49^*^
**Absolute weight of kidneys (g)**				
12	0.53 ± 0.07	0.52 ± 0.04	0.56 ± 0.08	0.70 ± 0.18^*^
24	0.55 ± 0.03	0.62 ± 0.12	0.57 ± 0.16	0.89 ± 0.05^*^
36	0.58 ± 0.06	0.65 ± 0.05	0.76 ± 0.15^*^	0.98 ± 0.18^*^
**Absolute weight of liver (g)**				
12	0.73 ± 0.16	0.73 ± 0.84	0.75 ± 0.04	0.87 ± 0.03^*^
24	0.74 ± 0.09	0.79 ± 0.078	0.78 ± 0.23	0.88 ± 0.08^*^
36	0.76 ± 0.18	0.80 ± 0.05	0.89 ± 0.11^*^	0.98 ± 0.22^*^

Values (means ± SE) bearing asterisk in a row significantly (p < 0.05) different compared with control group.

### The relative weight of visceral organs of *Labeo rohita*

Results showed that the relative liver weight of fish increased significantly (*p* < 0.05) at CuSO_4_ doses of 0.48 μgL^−1^ and 0.56 μgL^−1^ on experimental day 36th than the control group (Table 2). In contrast, the relative gills weight increased significantly (*p* < 0.05) on experimental days 24th and 36th in groups C (CuSO_4_: 0.48 μgL^−1^) and D (CuSO_4_: 0.56 μgL^−1^) compared to the control group. As far as the relative weight of the kidneys is concerned, that showed a significant (*p* < 0.05) increase on experimental days 12th, 24th, and 36th in group D (CuSO_4_: 0.56 μgL^−1^) and also in group C (CuSO_4_: 0.48 μgL^−1^) on experimental day 24 compared to control group (Table 3). Significantly (*p* < 0.05) relative weight of the brain increased on experimental days 12th, 24th, and 36th in groups C (CuSO_4_: 0.48 μgL^−1^) and D (CuSO_4_: 0.56 μgL^−1^) compared to the control group (Table 3).

**Table 3:** Relative weight of visceral organs of *Labeo rohita* treated with various concentrations of copper sulfate.

**Parameters/day**	**Groups/treatments**			
	**A (0.0)**	**B (0.28** μ**g.L**^−1^**)**	**C (0.42** μ**g.L**^−1^**)**	**D (0.56** μ**g.L**^−1^**)**
**Liver (g)**				
12	0.27 ± 0.03	0.27 ± 0.02	0.30 ± 0.03	0.31 ± 0.11
24	0.30 ± 0.05	0.28 ± 0.05	0.32 ± 0.04	0.35 ± 0.13
36	0.30 ± 0.03	0.31 ± 0.07	0.38 ± 0.11^*^	0.41 ± 0.12^*^
**Gills (g)**				
12	2.76 ± 0.26	3.14 ± 0.23	3.15 ± 0.28	3.23 ± 0.30
24	3.24 ± 0.25	3.07 ± 0.34	3.47 ± 0.53	3.81 ± 0.51^*^
36	3.19 ± 0.23	3.08 ± 0.27	3.93 ± 0.34^*^	3.97 ± 0.21^*^
**Kidneys (g)**				
12	0.34 ± 0.05	0.33 ± 0.03	0.33 ± 0.05	0.44 ± 0.12^*^
24	0.36 ± 0.03	0.38 ± 0.07	0.34 ± 0.12	0.45 ± 0.04^*^
36	0.35 ± 0.04	0.39 ± 0.03	0.48 ± 0.08^*^	0.49 ± 0.13^*^
**Brain (g)**				
12	0.32 ± 0.16	0.34 ± 0.16	0.46 ± 0.17^*^	0.51 ± 0.18^*^
24	0.36 ± 0.16	0.35 ± 0.01	0.55 ± 0.15^*^	0.58 ± 0.12^*^
36	0.34 ± 0.15	0.36 ± 0.13	0.58 ± 0.14^*^	0.59 ± 0.18^*^

Values (means ± SE) bearing asterisk in a row significantly (p < 0.05) different compared with control group.

### Hematological parameters

Different hematological parameters of fish exposed to several concentrations of CuSO_4_ are presented in Table 4. Results showed that RBC counts, hemoglobin, hematocrit, and lymphocytes decreased significantly (*p* < 0.05) while WBC counts and neutrophils increased significantly (*p* < 0.05) on experimental days 12, 24, and 36 in high dose group (CuSO_4_: 0.56 μg.L^−1^) compared to control group. In addition, hemoglobin and hematocrit decreased significantly (*p* < 0.05) at CuSO_4_ dose 0.42 μg.L^−1^ on experimental day 36 compared to the control group. WBCs counts also increased significantly (*p* < 0.05) on experimental day 24 and 36 at CuSO_4_ dose 0.42 μg.L^−1^ compared to control group (Table 4). MHC and MCHC in all treatment groups varied non-significant (*p* > 0.05) throughout the experiment as compared to the control group.

**Table 4:** Hematology of fish treated with various concentrations of copper sulfate.

**Parameters/day**	**Groups/treatments**			
	**A (0.0)**	**B (0.28** μ**g.L**^−1^**)**	**C (0.42** μ**g.L**^−1^**)**	**D (0.56** μ**g.L**^−1^**)**
**Red blood cell count (**×**10**^6^**/mm**^3^**)**				
12	4.83 ± 0.18	4.06 ± 0.19	3.96 ± 0.16	3.09 ± 0.05^*^
24	5.05 ± 0.29	4.03 ± 0.27	3.85 ± 0.03	3.33 ± 0.11^*^
36	5.02 ± 0.07	4.01 ± 0.11	3.83 ± 0.17	3.29 ± 0.18^*^
**Hemoglobin concentration (g.dL** ^−1^ **)**				
12	8.96 ± 0.23	8.73 ± 0.31	7.72 ± 0.28	6.91 ± 0.18^*^
24	9.03 ± 0.30	8.38 ± 0.47	7.69 ± 0.16	6.81 ± 0.26^*^
36	9.38 ± 0.20	8.68 ± 0.28	6.45 ± 0.13^*^	6.30 ± 0.21^*^
**White blood cell counts (**×**10**^3^**/mm**^3^**)**				
12	12.55 ± 0.21	15.09 ± 0.42	15.44 ± 0.51	16.67 ± 0.50^*^
24	13.01 ± 0.43	14.94 ± 0.22	16.28 ± 0.44^*^	18.47 ± 0.35^*^
36	12.56 ± 0.61	14.28 ± 0.42	17.22 ± 0.28^*^	18.96 ± 0.31^*^
**Hematocrit**				
12	34.14 ± 2.39	35.78 ± 0.94	29.53 ± 0.96	24.70 ± 0.58^*^
24	35.18 ± 0.41	34.73 ± 1.01	30.52 ± 0.95	23.48 ± 0.25^*^
36	36.50 ± 0.32	32.78 ± 0.36	26.35 ± 0.32^*^	23.30 ± 2.29^*^
**MCH (pg)**				
12	38.25 ± 0.28	35.88 ± 0.35	32.92 ± 1.05	14.48 ± 1.05
24	36.48 ± 0.96	36.55 ± 0.30	19.20 ± 1.72	15.94 ± 1.15
36	38.08 ± 0.89	37.43 ± 0.68	34.13 ± 1.64	17.95 ± 0.95
**MCHC (g.dL** ^−1^ **)**				
12	34.70 ± 0.67	33.25 ± 0.18	31 ± 0.86	26.02 ± 1.41
24	34.70 ± 0.82	32.85 ± 0.14	31.55 ± 0.44	25.53 ± 2.68
36	35.48 ± 0.50	33.93 ± 0.05	32.20 ± 0.88	24.98 ± 1.91
**Lymphocytes (%)**				
12	18.61 ± 0.54	17.50 ± 0.34	15.85 ± 0.18	14.85 ± 0.16^*^
24	20.34 ± 0.18	19.20 ± 0.37	16.79 ± 0.41	13.67 ± 0.18^*^
36	19.97 ± 0.36	18.35 ± 0.35	16.31 ± 0.29	12.75 ± 0.17^*^
**Neutrophils (%)**				
12	15.78 ± 0.22	17.37 ± 0.26	19.67 ± 0.65	22.50 ± 0.36^*^
24	15.97 ± 0.26	17.29 ± 0.29	19.78 ± 0.79	22.75 ± 0.43^*^
36	15.89 ± 0.23	17.35 ± 0.26	19.79 ± 0.69	22.62 ± 0.33^*^

Values (means ± SE) bearing asterisk in a row significantly (p < 0.05) different compared with control group.

### Serum biochemical parameters

Serum biochemical parameters of fish exposed to several concentrations of CuSO_4_ are presented in Table 5. The concentration of urea, creatinine, AST, LDH, cholesterol, glucose, and triglycerides increased significantly (*p* < 0.05) in the blood serum of fish treated with CuSO_4_ dose 0.56 μg.L^−1^ on experimental days 24 and 36 and also with CuSO_4_ dose 0.42 μg.L^−1^ on experimental day 36 compared to control group. In contrast, ALP and ALT increased significantly (*p* < 0.05) in fish treated with CuSO_4_ dose 0.56 μg.L^−1^ on experimental days 12, 24, and 36 and also with CuSO_4_ dose 0.42 μg.L^−1^ on experimental day 36 compared to control group. Total proteins and albumin concentration decreased significantly (*p* < 0.05) with the treatment of CuSO_4_ dose 0.56 μg.L^−1^ on experimental days 24 and 36, and total proteins also decreased significantly (*p* < 0.05) with the treatment of CuSO_4_ dose 0.42 μg.L^−1^ on experimental day 36 (Table 4).

**Table 5:** Serum biochemistry of fish treated to various concentrations of copper sulfate.

**Parameters/day**	**Groups/treatments**			
	**A (0.0)**	**B (0.28** μ**g.L**^−1^**)**	**C (0.42** μ**g.L**^−1^**)**	**D (0.56** μ**g.L**^−1^**)**
**Albumin quantity (mg.dL** ^−1^ **)**				
12	2.85 ± 0.01	2.81 ± 0.01	2.77 ± 0.01	2.73 ± 0.01
24	2.81 ± 0.01	2.77 ± 0.01	2.72 ± 0.01	2.18 ± 0.01^*^
36	2.79 ± 0.01	2.74 ± 0.01	2.70 ± 0.01	2.15 ± 0.01^*^
**Total proteins (mg.dL** ^−1^ **)**				
12	3.89 ± 0.03	3.72 ± 0.03	3.55 ± 0.03	3.38 ± 0.03
24	3.83 ± 0.03	3.63 ± 0.03	3.44 ± 0.03	3.14 ± 0.03^*^
36	3.75 ± 0.04	3.49 ± 0.04	3.14 ± 0.04^*^	2.08 ± 0.04^*^
**Aspartate aminotransferase (UL** ^−1^ **)**				
12	13.65 ± 0.06	15.05 ± 1.06	15.35 ± 0.06	15.75 ± 0.06
24	13.07 ± 0.09	15.55 ± 1.06	16.05 ± 0.06	17.52 ± 0.09^*^
36	13.80 ± 0.13	16.60 ± 0.13	17.30 ± 0.13^*^	18.10 ± 0.13^*^
**Alkaline phosphatase (UL** ^−1^ **)**				
12	24.55 ± 0.10	26.17 ± 0.11	26.82 ± 0.11	30.45 ± 0.10^*^
24	25.15 ± 0.10	26.82 ± 0.11	27.47 ± 0.11	32.15 ± 0.10^*^
36	24.40 ± 0.13	27.20 ± 0.13	29.10 ± 0.13^*^	33.90 ± 0.13^*^
**Alanine aminotransferase (UL** ^−1^ **)**				
12	22.05 ± 0.06	22.45 ± 0.06	22.75 ± 0.06	28.15 ± 0.06^*^
24	22.45 ± 0.10	23.07 ± 0.11	23.72 ± 0.11	29.35 ± 0.10^*^
36	23.10 ± 0.13	23.9 ± 0.13	29.80 ± 0.13^*^	32.60 ± 0.13^*^
**Lactate dehydrogenase (UL** ^−1^ **)**				
12	248.3 ± 2.19	249.5 ± 3.19	250.7 ± 1.19	251.9 ± 5.19
24	251.7 ± 1.48	254.6 ± 2.47	257.6 ± 2.47	269.6 ± 3.48^*^
36	253.3 ± 2.61	257.0 ± 3.61	268.7 ± 3.61^*^	274.4 ± 5.61^*^
**Urea (mg.L** ^−1^ **)**				
12	8.02 ± 0.04	8.57 ± 0.04	8.81 ± 0.04	9.06 ± 0.04
24	8.08 ± 0.05	8.87 ± 0.05	9.16 ± 0.05	10.45 ± 0.05^*^
36	8.04 ± 0.05	9.02 ± 0.05	10.29 ± 0.05^*^	11.57 ± 0.05^*^
**Creatinine (mg.L** ^−1^ **)**				
12	1.17 ± 0.01	1.18 ± 0.02	1.21 ± 0.02	1.23 ± 0.04
24	1.20 ± 0.04	1.22 ± 0.04	1.25 ± 0.04	1.87 ± 0.04^*^
36	1.28 ± 0.02	1.29 ± 0.02	1.72 ± 0.02^*^	1.94 ± 0.02^*^
**Cholesterol (mg.L** ^−1^ **)**				
12	154.6 ± 2.17	155.7 ± 3.17	156.8 ± 3.17	157.9 ± 2.17
24	156.5 ± 3.39	158.9 ± 3.39	161.2 ± 3.39	173.6 ± 2.39^*^
36	158.9 ± 1.50	162.1 ± 2.50	175.2 ± 2.50^*^	188.3 ± 3.50^*^
**Glucose (mg.L** ^−1^ **)**				
12	29.3 ± 1.16	30.2 ± 1.15	31.1 ± 1.15	32.1 ± 2.16
24	29.6 ± 1.19	30.8 ± 1.19	32.2 ± 1.19	35.2 ± 1.19^*^
36	30.7 ± 1.19	31.9 ± 1.19	35.6 ± 1.19^*^	37.4 ± 1.19^*^
**Triglycerides (mg.L** ^−1^ **)**				
12	151.6 ± 1.34	157.7 ± 2.32	159.8 ± 1.32	160.9 ± 2.34
24	153.2 ± 2.35	158.4 ± 2.35	161.6 ± 1.35	179.8 ± 3.35^*^
36	156.3 ± 3.41	159.7 ± 2.41	181.2 ± 2.41^*^	183.7 ± 2.41^*^

Values (means ± SE) bearing asterisk in a row significantly (p < 0.05) different compared with control group.

### Oxidative stress and antioxidant enzymes

The results of oxidative stress (Thio-barbituric acid reactive substances) and different antioxidant enzymes (reduced glutathione, superoxide dismutase, and catalase) are presented in Table 6. Results recorded a significantly (*p* < 0.05) increased quantity of TBARS in fish treated with CuSO_4_ dose 0.56 μg.L^−1^ on experimental days 24 and 36 and with CuSO_4_ dose 0.42 μg.L^−1^ on experimental day 36 compared to the control group. The TBARS also increased significantly (*p* < 0.05) with the treatment of CuSO_4_ dose 0.42 μg.L^−1^ on experimental day 36 (Table 5). There were significantly (*p* < 0.05) decreased concentrations of different antioxidant enzymes, including reduced glutathione, superoxide dismutase, and catalase in erythrocytes of fish treated with CuSO_4_ dose 0.56 μg.L^−1^ on experimental days 24 and 36 and also with CuSO_4_ dose 0.42 μg.L^−1^ on experimental day 36 compared to control group (Table 6).

**Table 6:** Oxidative stress and antioxidant enzymes in erythrocytes of fish (*Labeo rohita)* treated to copper sulfate.

**Parameters/day**	**Groups/treatments**			
	**A (0.0)**	**B (0.28** μ**g.L**^−1^**)**	**C (0.42** μ**g.L**^−1^**)**	**D (0.56** μ**g.L**^−1^**)**
**Thio-barbituric acid reactive substances (TBARS) contents (nmol TBARS/h/mg protein)**				
12	37.21 ± 1.11	38.41 ± 1.13	40.13 ± 1.04	41.25 ± 1.17
24	38.71 ± 1.21	39.21 ± 1.11	40.89 ± 1.37	46.35 ± 1.33^*^
36	39.31 ± 2.01	41.71 ± 1.28	47.31 ± 1.55^*^	53.19 ± 166^*^
**Reduced glutathione (**μ**mol GSH/mg protein)**				
12	0.12 ± 0.04	0.11 ± 0.03	0.11 ± 0.01	0.19 ± 0.03
24	0.13 ± 0.01	0.12 ± 0.08	0.10 ± 0.07	0.08 ± 0.01^*^
36	0.13 ± 0.02	0.11 ± 0.06	0.07 ± 0.01^*^	0.06 ± 0.02^*^
**Superoxide dismutase (units/mg protein)**				
12	2.33 ± 0.19	2.19 ± 0.13	1.99 ± 0.17	1.88 ± 0.11
24	2.37 ± 0.15	2.11 ± 0.15	1.88 ± 0.11	1.64 ± 0.05^*^
36	2.38 ± 0.15	2.03 ± 0.16	1.45 ± 0.21^*^	1.39 ± 0.11^*^
**Catalase (units/mg protein)**				
12	2.45 ± 0.07	2.43 ± 0.09	2.39 ± 0.13	2.33 ± 0.15
24	2.47 ± 0.14	2.35 ± 0.23	2.22 ± 0.21	1.68 ± 0.05^*^
36	2.46 ± 0.12	2.25 ± 0.16	1.71 ± 0.02^*^	1.37 ± 0.22^*^

Values (means ± SE) bearing asterisk in a row significantly (p < 0.05) different compared with control group.

### Histological alterations in various tissues

**Figure 1:**
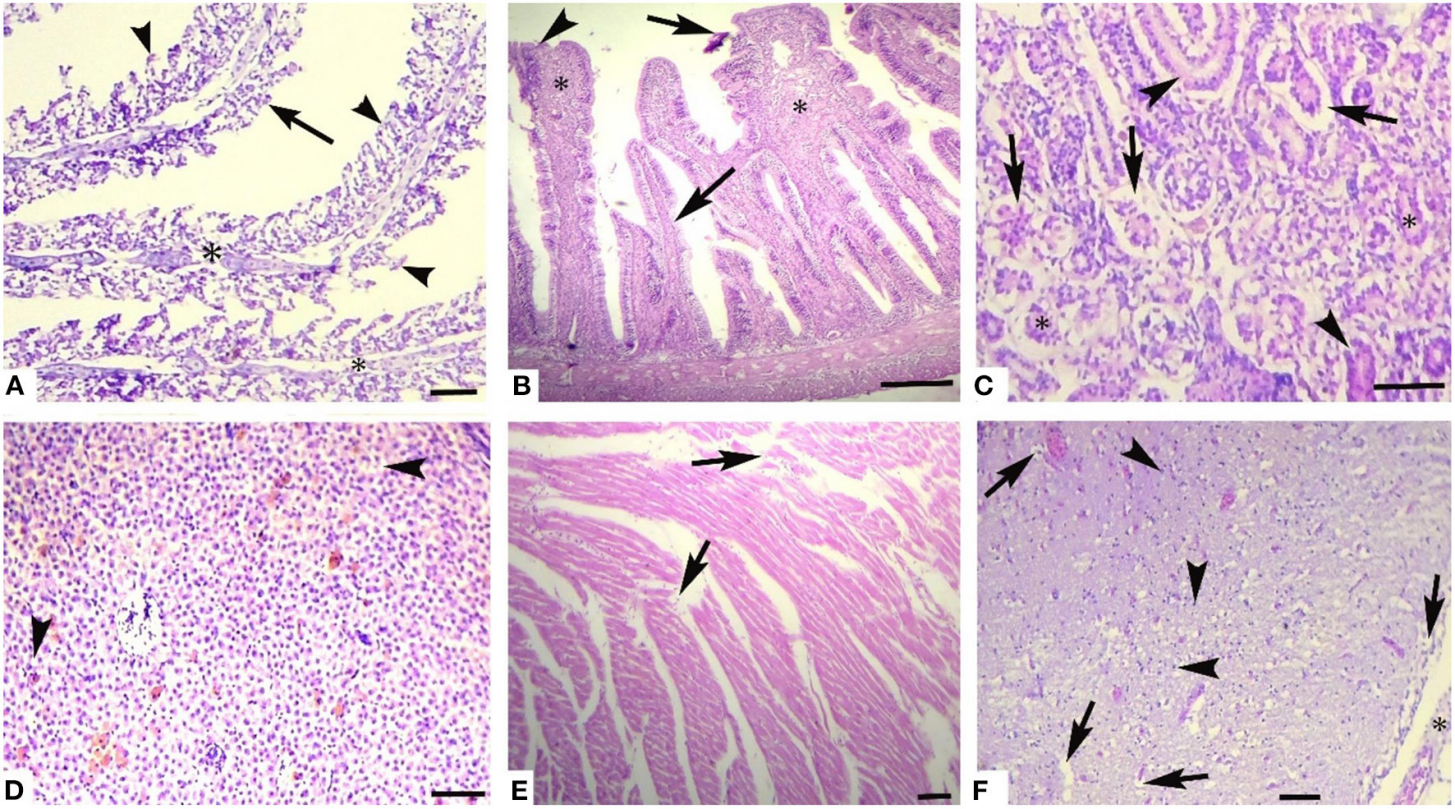
Photomicrograph of different tissues of freshwater fish treated with CuSO_4_ (0.42 μg.L^−1^) at experimental day 36: **(A)** Gills showing aneurysm (arrowheads), disorganization of primary lamellae (double asterisk) and necrosis of epithelium (arrows), **(B)** Intestine showing sloughing of epithelium from villi (arrows), necrosed epithelium (arrowheads) and necrosis of middle portio of villi (asterisk), **(C)** kidneys showing detachment of epithelium from the basement membrane (arrows), necrosis of renal tubules epithelial cells (arrowheads), protenecious material in tubulat lumen (asterisk) **(D)** Liver showing vacuolar degeneration (arrowheads) and necrosis of hepatocytes, **(E)** Heart showing disorganization of cardiac muscles (arrow), and **(F)** Brain showing necrosis of neurons (arrowheads), vacuolar degeneration of neurons (arrows) and microgliosis (asterisk). H and E. Bar is equal to **(A)** and **(E)** = 50 μm, and **(B–D)** and **(F)** = 100 μm.

**Figure 2:**
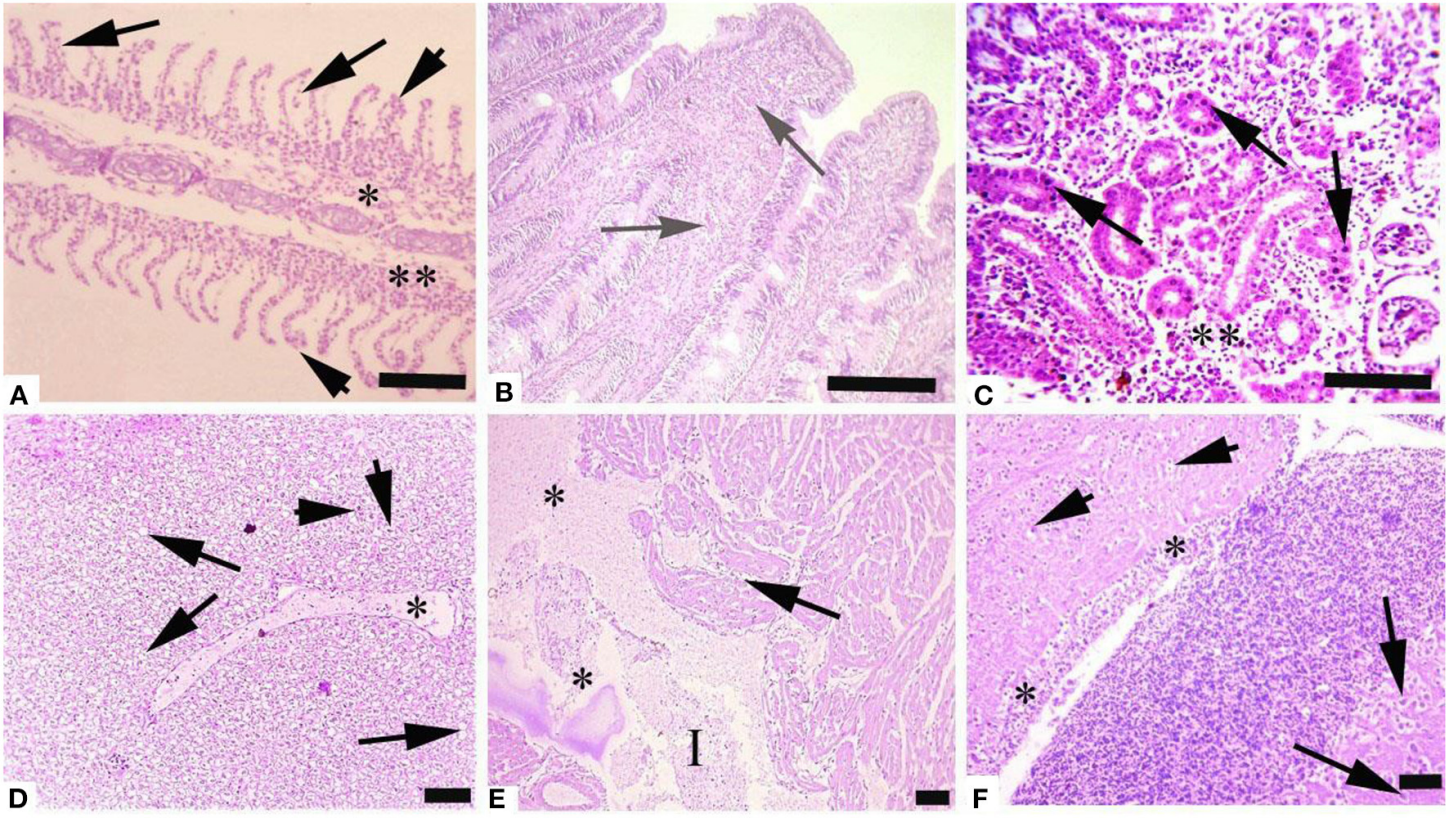
Photomicrograph of different tissues of freshwater fish treated with CuSO_4_ (0.56 μg.L^−1^) at experimental day 36: **(A)** Gills showing aneurysm (arrowheads), disorganization of primary lamellae (double asterisk) and necrosis of epithelium of secondary lamellae (arrows), **(B)** Intestine showing hemorrhages (arrows), **(C)** kidneys showing necrosis of renal tubules (asterisk) and necrosis of tubular epithelial cells (arrows), **(D)** Liver showing edema (asterisk), vacuolar degeneration (arrows) and necrosis (arrowheads), **(E)** Heart showing disorganization of cardiac muscles (arrow), edema (asterisks) and inflammatory exudate (I) and **(F)** Brain showing necrosis of neurons (arrowheads), degeneration of neurons (arrows) and microgliosis (asterisk). H and E. Bar is equal to **(A)** and **(E)** = 50 μm, and **(B–D)** and **(F)** = 100 μm.

Histological investigation of the liver, gill, kidneys, and brain tissues on experimental days 12, 24, and 36 of the control and copper sulfate treated fish was carried out by the use of H and E staining, and various alterations in the tissue morphology were observed and presented below in Tables 7–9 and Figures 1, 2. Dose and time-dependent histopathological alterations in kidneys, gill, liver, and brain tissues occurred; those were fewer at CuSO_4_ dose 0.28 μg.L^−1^ and experimental day 12 and increased significantly (*p* < 0.05) with increasing the dose of CuSO_4_ from 0.42 μg.L^−1^ to 0.56 μg.L^−1^ and experimental day from 24 to 36. The degree of histopathological lesions was moderate to severe (Figure 1) at experimental day 36 with CuSO_4_ dose 0.42 μg.L^−1^ whereas these were severe to very severe (Figure 2) at CuSO_4_ dose 0.56 μg.L^−1^ at day 36. The Control group did not exhibit any histopathological alterations.

**Table 7:** Severity/degree of different histopathological lesions in various organs/tissues of *Labeo rohita* treated to various concentrations of copper sulfate at 12th day.

**Histopathological lesions**	**Groups**		
	**B (0.28** μ**g.L**^−1^**)**	**C (0.42** μ**g.L**^−1^**)**	**D (0.56** μ**g.L**^−1^**)**
**Brain**			
Congestion	++	+++	++++
Neuron's necrosis	++	+++	++++
Intracellular edema	+	++	+++
Cytoplasmic vacuolization	+	++	++++
**Gills**			
Congestion	++	+++	++++
Lamellar pillar cell's necrosis	++	+++	++++
Cartilaginous core congestion	++	+++	++++
Cartilaginous core degeneration	+	++	+++
Lamellar fusion	+	++	++++
Lamellar atrophy	+	+++	++++
Lamellar disorganization	+	+++	++++
Telangiectasia	++	+++	++++
Lamellae uplifting	+	+++	++++
Primary lamellae disruption	+	++	++++
Secondary lamellae curling	+	++	+++
**Liver**			
Congestion	++	+++	++++
Vacuolar degeneration	+	++	+++
Hemorrhages	++	+++	++++
Hepatocytes with eccentric nuclei	+	+++	++++
Hepatocyte degeneration	++	++	+++
Pyknosis	+	+++	++++
Karyorrhexis	+	++++	++++
Karyolysis	+	+++	++++
Nuclear hypertrophy	++	++	++++
Ceroid formation	++	+++	++++
**Kidneys**			
Ceroid formation	++	+++	++++
Congestion	++	++	+++
Edema	++	++	+++
Bowman's space increased	+	++	+++
Tubular cells necrosis	+	++	+++
Glomerulus deterioration	+	++	+++
Renal tubule degeneration and abolition	++	+++	++++
Nuclear hypertrophy	++	+++	++++
Melanomacrophage aggregates	++	+++	++++
Thyroidization	++	+++	++++

The control group did not show any lesion; thus was not mentioned in the Table. The degree of histopathological lesions was based on severity as mild (+, 25%), moderate (++, 50%), severe (+++, 75%) and very severe (++++, 100%).

**Table 8:** Severity/degree of different histopathological changes in various tissues of *Labeo rohita* exposed to various concentrations of copper sulfate at day 24th.

**Histopathological lesions**	**Groups**		
	**B (0.28** μ**g.L**^−1^**)**	**C (0.42** μ**g.L**^−1^**)**	**D (0.56** μ**g.L**^−1^**)**
**Brain**			
Congestion	++	+++	++++
Neuron's necrosis	++	+++	++++
Intracellular edema	++	+++	++++
Cytoplasmic vacuolization	+	++	++++
**Gills**			
Congestion	++	+++	++++
Lamellar pillar cell's necrosis	++	+++	++++
Cartilaginous core congestion	++	+++	++++
Cartilaginous core degeneration	+	++++	++++
Lamellar fusion	+	++++	++++
Lamellar atrophy	+	++++	++++
Lamellar disorganization	++	+++	++++
Telangiectasia	++	+++	++++
Lamellae uplifting	++	++++	++++
Primary lamellae disruption	++	++++	++++
Secondary lamellae curling	++	+++	++++
**Liver**			
Congestion	++	++	++++
Vacuolar degeneration	+	+++	++++
Hemorrhages	++	+++	++++
Hepatocytes with eccentric nuclei	++	++++	++++
**Hepatocyte degeneration**			
Pyknosis	+	+++	++++
Karyorrhexis	+	++++	++++
Karyolysis	++	++	++++
Nuclear hypertrophy	++	+++	++++
Ceroid formation	+	++	++++
**Kidneys**			
Ceroid formation	++	++	++++
Congestion	++	+++	++++
Edema	++	+	++++
Bowman's space increased	++	+++	++++
Tubular cells necrosis	++	+++	++++
Glomerulus deterioration	++	+++	++++
Renal tubule degeneration and abolition	+	++	++++
Nuclear hypertrophy	++	++	++++
Melanomacrophage aggregates	+	++	++++
Thyroidization	++	++	++++

The control group did not show any lesion; thus was not mentioned in the Table. The degree of histopathological lesions was based on severity as mild (+, 25%), moderate (++, 50%), severe (+++, 75%) and very severe (++++, 100%).

**Table 9:** Severity/degree of different histopathological changes in various tissues of *Labeo rohita* exposed to different concentrations of copper sulfate at day 36.

**Histopathological lesions**	**Groups**		
	**B (0.28** μ**g.L**^−1^**)**	**C (0.42** μ**g.L**^−1^**)**	**D (0.56** μ**g.L**^−1^**)**
**Brain**			
Congestion	++	+++	++++
Neuron's necrosis	++	+++	++++
Intracellular edema	++	+++	++++
Cytoplasmic vacuolization	+	++	++++
**Gills**			
Congestion	++	+++	++++
Lamellar pillar cell's necrosis	++	+++	++++
Cartilaginous core congestion	++	+++	++++
Cartilaginous core degeneration	+	++++	++++
Lamellar fusion	+	++++	++++
Lamellar atrophy	+	++++	++++
Lamellar disorganization	++	+++	++++
Telangiectasia	++	+++	++++
Lamellae uplifting	++	++++	++++
Primary lamellae disruption	++	++++	++++
Secondary lamellae curling	++	+++	++++
**Liver**			
Congestion	++	+++	++++
Vacuolar degeneration	++	+++	++++
Hemorrhages	++	+++	++++
Hepatocytes with eccentric nuclei	++	+++	++++
Hepatocyte degeneration	++	+++	++++
Pyknosis	+	+++	++++
Karyorrhexis	++	++++	++++
Karyolysis	++	+++	++++
Nuclear hypertrophy	++	+++	++++
Ceroid formation	++	+++	++++
**Kidneys**			
Ceroid formation	++	+++	++++
Congestion	++	+++	++++
Edema	++	+++	++++
Bowman's space increased	++	+++	++++
Tubular cells necrosis	++	+++	++++
Glomerulus deterioration	++	+++	++++
Renal tubule degeneration and abolition	++	++++	++++
Nuclear hypertrophy	++	+++	++++
Melanomacrophage aggregates	+	+++	++++
Thyroidization	++	++	++++

The control group did not show any lesion; thus was not mentioned in the Table. The degree of histopathological lesions was based on severity as mild (+, 25%), moderate (++, 50%), severe (+++, 75%) and very severe (++++, 100%).

## Discussion

Copper sulfate has been reported to cause deleterious effects at higher levels (53–55). Sometimes the water quality, like pH, dissolved organic carbon, and hardness, also affect the toxicity of metal (e.g., Cu, Zn, Ni) in aquatic environments (22). Among all the environmental issues, metal pollution is the most serious and major hazard to the safety of humans and worldwide ecosystems (21, 44, 56–58).

The current study showed that various concentrations of CuSO_4_ affect fish health in several ways. In our trial, we observed no mortality in both control and CuSO_4_-treated experimental groups. However, the treated groups showed different clinical and behavioral signs like rapid operculum movement, tremors of fins, hypersecretion of mucus, erratic swimming, increased swimming area, loss of equilibrium, loss of coordination, increased surface breathing, and air gulping. Though no mortality was recorded in the present study, however high survival rate in the Japanese Medaka (*Oryzias latipes*) fish has been reported with exposure to low (10 BBP) compared with high (100 BBP) dose of CuSO_4_ (59). In the present study, the relative and absolute weight of fish exposed to CuSO_4_ showed a significant (*p* < 0.05) reduction in the treated groups compared to the control group. Similarly, it has been reported that *Epinephelus coioides* supplemented with CuSO_4_ and copper nanoparticles (Cu-NPs) rearing water exhibited lower weight gain than the control group (60). A decrease in growth performance could be due to two factors: in homeostasis and detoxification, metabolic expenditure increases, and higher exposure to Cu reduces feed intake, which ultimately leads to growth reduction (61).

In the present trial, various hematological and histopathological parameters were studied. Hematological signs are important for environmental monitoring, toxicological research, and indicators for many diseases. These parameters are critical for determining the physiological condition of fish under stress caused by metals or contaminants. In the current study, hematological parameters such as WBCs, and neutrophils were increased with the increase in concentration and duration. However, erythrocytes (4.83 ± 0.18 vs. 3.09 ± 0.05 × 10^6^/mm^3^), hematocrit (34.14 ± 2.39 vs. 24.70 ± 0.58 %), and hemoglobin (8.96 ± 0.23 vs. 6.91 ± 0.18 g.dL^−1)^, and lymphocytes (18.61 ± 0.54 vs. 14.85 ± 0.16 %) were decreased significantly (*p* < 0.05) after the exposure to CuSO_4_ (0.28 μg.L^−1^) on all experimental days. Similarly, CuSO_4_ (0.05 mg/L) exposure to fish *Oreochromis niloticus* had been reported to significantly (*p* < 0.05) decrease erythrocytes (1.78 ± 0.03 vs. 1.41 ± 0.02 10^6^/μL), hematocrit (31.95 ± 0.74 vs. 24.83 ± 0.37 %), and hemoglobin (8.38 ± 0.09 vs. 5.94 ± 0.12 g/dL) values (17). It is also on record that CuSO_4_-NPs render more reduction in these parameters than CuSO_4_ itself (17). The lower hematological values in this study might be due to hemoglobin oxidation, erythrocyte destruction, and hemolysis (23, 47, 53, 56). Previous studies also described a decline in RBCs, Hb, and Hct contents in freshwater fishes exposed to Cd and Ni (58). In the current study, the hematological abnormalities might be due to the destruction or decreased production of RBCs in hematopoietic tissues, increased production of free radicals, and insufficient supply of oxygen to blood-forming tissues through gills. Moreover, several other studies have reported similar lower hematological values in different organisms like albino rats (62), yellow catfish (61), freshwater fish (47), and *Catla Catla* (23) exposed to various toxicants or pollutants. Additionally, the increased WBCs values and neutrophil percentage could be related to the stress conditions induced by the inflammatory response.

In the present study, except total proteins (3.75 ± 0.04 vs. 2.08 ± 0.04 mg.dL^−1^), all biochemical parameters including serum urea (8.04 ± 0.05 vs.11.57 ± 0.05 mg.L^−1^), creatinine (1.28 ± 0.02 vs. 1.94 ± 0.02 mg.L^−1^), cholesterol (158.9 ± 1.50 vs. 188.3 ± 3.50 mg.L^−1^), glucose (30.7 ± 1.19 vs. 37.4 ± 1.19 mg.L^−1^), triglycerides (156.3 ± 3.41 vs.183.7 ± 2.41 mg.L^−1^) along with serum enzymes i.e., ALT (23.10 ± 0.13 vs. 32.60 ± 0.13 UL^−1^), AST (13.80 ± 0.13 vs.18.10 ± 0.13 UL^−1^), ALP (24.40 ± 0.13 vs. 33.90 ± 0.13 UL^−1^) and LDH (253.3 ± 2.61 vs. 274.4 ± 5.61 UL^−1^) increased significantly (*p* < 0.05) in *L. rohita* with the exposure of CuSO_4_ (Table 5). However, along with other biochemical parameters such as glucose (105 ± 1.1 vs. 115 ± 0.5 mg/dL), AST (55.8 ± 0.3 vs. 57.4 ± 0.2 μg/L), ALT (28.6 ± 0.4 vs. 30.8 ± 0.5 μg/L) and uric acid (12.7 ± 0.04a 13 ± 0.1 mg/dL), total proteins (5.6 ± 0.1 vs. 6.4 ± 0.1 g/dL) also increased significantly (*p* < 0.05) in Nile tilapia (*Oreochromis niloticus*) with the treatment of both CuSO_4_ (15 mg/L) and CuO-NP (24). Increased total proteins in Nile tilapia with the treatment of CuSO_4_ (24) could be the reason that during exposure to Cu, fish may require higher protein concentration to meet the increased demand for repairing tissue and enhance the immunological response (20). Similarly, Atta et al. (63) reported that birds supplemented with excessive CuSO_4_ revealed an increase in ALP, ALT, and AST activities while reducing albumin, globulin levels, and total serum protein are consistent with our results. The hypoproteinemia in this study could be due to impairment of protein synthesis or the functional damage to the liver (31), and excessive loss of protein caused by degenerated glomerulus or could be explained due to the oxidative stress of copper on the liver and kidney tissue (64). There is also the possibility that the release of intracellular enzymes as a result of hepatic degeneration and necrosis caused by CuSO_4_ could lead to lipid peroxidation, which resultantly ends in hypoproteinemia (65). In the present research, the cholesterol levels increased significantly in *L. rohita* exposed to CuSO_4_ in a dose-dependent manner. Similarly, the findings of Almansour (66) are consistent with our results, who observed an increase in the cholesterol, total lipid, and LDL with no variation in HDL level in quail that were exposed to copper. Contrarily, no alteration was seen in total plasma cholesterol levels in broilers exposed to CuSO_4_ (67).

In this experimental trial, TBARS (39.31 ± 2.01 vs. 53.19 ± 166 nmol TBARS/h/mg protein) increased significantly (*p* < 0.05) whereas RGSH (0.13 ± 0.02 vs. 0.06 ± 0.02 μmol GSH/mg protein), SOD (2.38 ± 0.15 vs. 1.39 ± 0.11 units/mg protein) and CAT (2.46 ± 0.12 vs. 1.37 ± 0.22 units/mg protein) decreased significantly (*p* < 0.05) in red blood cells of *L. rohita* with the exposure of CuSO_4_ (Table 6). It has been reported that antioxidant enzymes such as SOD (11.6 ± 0.1 vs. 12.1 ± 0.13 IU/L), TAC (1.1 ± 0.01 vs. 0.9 ± 0.02 μM/L) and CAT (10.7 ± 0.1 vs. 11.5 ± 0.1 IU/L) increased significantly (*p* < 0.05) in Nile tilapia (*Oreochromis niloticus*) with the treatment of both CuSO_4_ (15 mg/L) and CuO-NP (24). The reduced quantity of different oxidant enzymes in erythrocytes of CuSO_4_ treated fish could be due to higher turnover of free radicals leading to depletion of antioxidants enzymes and induction of oxidative stress (40, 55, 68–70). Reduction in different antioxidant enzymes, including SOD, CAT, POD, and RGSH in erythrocytes of fish can possibly be due to different physiological disorders in erythrocytes and poor integrity of erythrocytic membranes in association with induction of low-grade inflammatory mechanisms in treated fish leading to inhibition of release of TNF-α and IL-1 (71). Studies have recorded that monitoring and evaluation of oxidative stress are of vital importance, which plays a vital role in the development of different physiological disorders in animals and also leads to lipid peroxidation, DNA damage, apoptosis, enzymic deactivation, and cell necrosis (71–75).

Histopathological changes have been extensively utilized as biomarkers while assessing the health of exposed fish to toxins/pollutants (76). Moreover, histopathology is the microscopic assessment of alteration in morphology that indicates disease in an organism (77, 78). We observed histopathological alterations in liver tissues, like ceroid formation, congestion, vacuolar degeneration, hemorrhages, pyknosis, karyorrhexis, degenerated hepatocytes, hepatocytes with eccentric nuclei, and karyolysis. While in kidney tissues, histopathological changes like widened Bowman's space, congestion, necrosis of tubular cells, edema, ceroid formation, melano-macrophage aggregates, glomerulus deterioration, thyroidization, and degeneration of kidney tubules were observed in *L. rohita* exposed to copper sulfate. These changes were dose-dependent.

The liver is considered as cutting edge immune organ (79). CuSO_4_ may possibly accrue in the liver. Liver is involved in the detoxification of metals at cellular level (80). Continual exposure to copper sulfate could have many detrimental effects on living beings (81). Copper have a tendency to bioaccumulate, disturbing human/animals food chain and eventually life menacing. Overexposure to copper generates inflammatory response, and oxidative stress, thus leading to liver injury (82–84).

Copper sulfate produces multiorgan (lungs, liver, gastrointestinal tract, and kidneys) injury/damage leading to disfunction *via* ROS production rendering oxidative stress (85). In hepatotoxicity, a vicious circle between oxidative stress and inflammatory response has been reported (86). Direct effect of copper usually leads to DNA and cell damage which then results in oxidative stress or enhanced discharge of ROS due to stimulation of phagocytic cells and increased tissue damage (6, 40, 70, 72, 87) as have been observed at high doses of CuSO_4_ in the present study. Moreover, endoplasmic reticulum and oxidative stress leads to nephrotoxicity as a result of CuSO_4_-exposure (5). Injury to various organs may result in fish mortality (88). Copper sulfate toxicity usually causes degeneration in renal tubules and glomerulus, and hepatocytes necrosis. These types of lesions hamper kidney and liver functions (89–91) which result in elevated enzymes such as LDH, ALT, and AST that has been seen in the present study.

Similarly, Abdel-Latif et al. (92) conducted a study on Nile tilapia. They found that exposure to sub-lethal concentration of CuO-NPs resulted in necrosis, hepatocytes degeneration, and severe congestion of blood sinusoids corresponding to the exposure dose, as well as increased Bowman's spaces, pyknotic nuclei, necrosis of kidney tubules, multi-focal inter-tubular hemorrhages and renal edema.

The gills are the first organ affected by exposure to CuSO4 in water (81). In the current study, histopathological alterations of gill tissues such as congestion of the cartilaginous core, congestion, telangiectasia, necrosis of lamellar pillar cells, lamellar fusion, lamellar atrophy, cartilaginous core degeneration, primary lamellae disruption, uplifting of lamellae, and disorganization of lamellar were observed, while in brain tissue degenerative changes like neurons necrosis, intracellular edema, cytoplasmic vacuolization, and congestion were observed in the studied fish at different exposure intervals during the experiment, which increases with an increase in concentration. Previously it has been indicated edematous changes in the gills could possibly due increased capillary permeability (88). Copper sulfate causes hyperplasia of gills epithelial cells, and it is dose dependent (93, 94) and it also leads to primary and secondary lamellar epithelial cells degeneration (76). Hyperplasia of gills epithelium along with degeneration and complete to partial lamellar fusion could be responses of the gills to pollutants (95, 96). Hyperplasia with thickening of gill filament epithelium may lead to the lamellar fusion (97). Augmented absorbency of the gill capillary walls subsequent vessel distension at the site of toxicant injury might be liable for the noted lamellar edema (98). There is another possibility that hyperplasia of gills could be a defensive aspect against irritants/heavy metals by reducing the mucosal membrane of respiratory tract and increasing the distance between toxicant–blood dissemination and its escalation which could result in the thickness of epithelial layers that could support by the intensifications of lamellar width and epithermus. Hence, lesions found in the current study could possibly impede the secretory, excretory, and respiratory functions of fish gills (81, 88, 99).

In the present study, CuSO_4_ dose and time-dependent histopathological alterations in brain tissues including congestion, cytoplasmic vacuolization, intracellular edema, and neuron necrosis were recorded. In the published literature, CuSO_4_ or CuO-NPs post disclosure, neuropil degeneration was obvious in Nile tilapia exposed to copper (24). These lesions could have developed as copper can cross blood-brain barrier (100–102). Several other studies (103–105) have reported similar lesions in brain of in response to various toxic chemicals and pollutants. Histopathological lesions in brain of *L. rohita* (106), *O. punctatus* (107), *C. gariepinus* (103), *C. catla* (108), *C. Carpio* (104), and *O. mossambicus* (97, 105) have been reported due to several toxicants exposure.

## Conclusion

Many changes in the hematological and serum biochemical parameters and histopathological status in *Labeo rohita* were observed in the current study. These changes directly affect the health status of fish and all other aquatic organisms exposed to these pollutants/chemicals/compounds. This study observed that exposure to even a low concentration of CuSO_4_ (0.28 μg.L^−1^) might result in physiological and histopathological alterations in multiple fish tissues. Based on the results derived from this study, further studies are proposed to find out the more suitable dose of CuSO_4_ for regular use to decontaminate fish ponds. It is also suggested that due to possible effects on the ecosystem, aquatic organisms, and animal and human health, we should regularly monitor exposure to CuSO4 in waters and continuously observe the environment/ecosystem. Hence, we must prioritize studies about environmental monitoring of pollutants and their possible effects on different organisms.

## Novelty of the study

While studying copper sulfate toxicity in fish in the past, people have reported mostly hematological or biochemical parameters singly or in combination. Whereas, in this study, we have reported comprehensively and simultaneously, physical, hematological, or biochemical, oxidative stress parameters along with gross and histopathology of fish exposed to various doses and at various time intervals.

## Data availability statement

The raw data supporting the conclusions of this article will be made available by the authors, without undue reservation.

## Ethics statement

The animal study was reviewed and approved by Bioethics Committee, The Islamia University Bahawalpur, Pakistan.

## Author contributions

SN and RH had the initial idea for the study. They were involved in the experiment, laboratory, data analysis, writing and development of the article, and the final proof stage. RH, SJ, and AC were involved in experimental studies, laboratory studies, and the article's writing. ML was involved in experimental studies, laboratory studies, and data analysis. ZG and AK performed and interpreted the statistical analysis. ZR arranged the facilities (enzymes, reagents). AK valuable suggestions regarding histopathological studies. All authors have read and approved the final version of the manuscript.
